# Abdominal Obesity Is Associated with an Increased Risk of All-Cause Mortality in Males but Not in Females with HFpEF

**DOI:** 10.1155/2022/2950055

**Published:** 2022-04-09

**Authors:** Liyao Fu, Ying Zhou, Jiaxing Sun, Zhaowei Zhu, Shi Tai

**Affiliations:** ^1^Department of Pathophysiology, Xiangya School of Medicine, Central South University, Changsha, Hunan, China; ^2^Department of Blood Transfusion, The Second Xiangya Hospital of Central South University, Changsha, China; ^3^Department of Cardiovascular Medicine, The Second Xiangya Hospital of Central South University, Changsha, China

## Abstract

**Background:**

Association between abdominal obesity and development of heart failure (HF) with preserved ejection fraction (HFpEF) between the sexes is not completely understood.

**Objectives:**

This study evaluated the association between abdominal obesity and the risk of all-cause mortality in patients with HFpEF while performing a gender outcome comparison.

**Methods:**

A post hoc analysis was undertaken from the American cohort of the Treatment of Preserved Cardiac Function Heart Failure with an Aldosterone Antagonist (TOPCAT). The primary outcome (all-cause mortality) and the secondary outcomes (cardiovascular mortality, hospitalization for HF, stroke, and MI) were evaluated via Cox proportional hazards models to compare the hazard ratios (HRs) between sexes in HFpEF patients. Abdominal obesity was defined as a waist circumference of ≥102 cm in men and ≥88 cm in women.

**Results:**

A total of 3320 HFpEF patients (1620 men [48.80%] and 1700 women [51.20%]) were included in the analysis. The mean follow-up period was 3.4 ± 1.7 years, with 503 patients dying during that time. After multivariable adjustment, abdominal obesity was significantly associated with an increased risk of all-cause mortality in males (adjusted HR: 1.32; 95% confidence interval [CI]: 1.02 to 1.71; *p* = 0.038). Abdominal obesity was associated with hospitalization for HF in both male (adjusted HR: 1.39; 95% CI: 1.01 to 1.93; *p* = 0.045) and female patients (adjusted HR: 1.15; 95% CI: 1.18 to 3.28; *p* = 0.010).

**Conclusions:**

Abdominal obesity is associated with increased risks of all-cause mortality in the male but not the female HFpEF population and is associated with increased risks of hospitalization for HF in both sexes.

## 1. Introduction

Heart failure (HF) is a clinical syndrome that affects millions of individuals globally. It is associated with a significant risk of mortality for patients and levies a heavy cost on society [1]. HF with preserved ejection fraction (HFpEF) is the most common type of HF and accounts for approximately half of all cases. This number is only expected to increase in the upcoming decades [2]. Patients diagnosed with HFpEF display the typical symptoms of HF (dyspnea, fatigue, intolerance to the effort, and peripheral edema), normal or mildly reduced ejection fraction (EF ≥50%), left atrial enlargement and/or diastolic dysfunction, and left ventricular (LV) hypertrophy [1, 3]. There are no approved therapies to reduce hospitalizations or mortality in HFpEF patients, and clinical guidelines have offered no specific recommendations for its management.

The pathophysiology of HFpEF, as a multifactorial disease, is both complex and poorly understood. Proinflammatory conditions, cardiac hypertrophy, arterial and microvascular dysfunction, impaired systolic and diastolic function, and interstitial cardiac fibrosis have been shown as systemic triggers for HFpEF [4]. Patients with HFpEF frequently present noncardiac comorbidities: obesity (prevalence of 84%), arterial hypertension (60%–80%), diabetes (20%–45%), chronic kidney disease (prevalence varies depending on definition; ~20–30%), sleep apnea, chronic obstructive pulmonary disease (COPD), and anemia [5–7]. These comorbidities can cause low-grade systemic inflammation and promote microvascular dysfunction and cardiomyocyte remodeling, which often results in left ventricular dysfunction [8]. A growing number of studies show that abdominal obesity is a significant contributor to systemic inflammation leading to myocardial remodeling, with a resultant HFpEF [5, 8]. Abdominal obesity, especially common in HFpEF, is the focus of this research. Notably, gender differences are related to body fat distribution, obesity prevalence, and energy homeostasis [9, 10]. Furthermore, increasing evidence indicates that there are gender differences in the correlation between abdominal obesity and the risk of cardiovascular disease (CVD) [11]. In this context, it can be suggested that abdominal obesity could influence the prognosis of HFpEF, with variations between the sexes. However, the role of underlying gender differences in abdominal obesity and cardiometabolic dysfunction remains unknown. This study aims to explore the impact of abdominal obesity on the prognosis of HFpEF and compare it between genders.

## 2. Methods

### 2.1. Study Population

This retrospective study utilized data from the TOPCAT (Treatment of Preserved Cardiac Function Heart Failure with an Aldosterone Antagonist) trial, which was a phase 3, international, multisite, double-blind, randomized, placebo-controlled trial. TOPCAT enrolled 3445 patients from 6 countries: the United States, Canada, Russia, Republic of Georgia, Argentina, and Brazil, from August 10, 2006, to January 31, 2012. Participants were randomly assigned to receive spironolactone or a placebo. This study was sponsored by the US National Heart, Lung, and Blood Institute. The design, protocol, and patient characteristics of the TOPCAT study have been previously reported [12]. The study presented here was approved by the Human Research Committee of The Second Xiangya Hospital of Central South University before study onset.

### 2.2. Data Collection and Outcomes

All demographic, clinical, and laboratory data were obtained from the trial database, which had been collected from the National Heart, Lung, and Blood Institute's Biologic Specimen and Data Repository Information Coordinating Center (BioLINCC). The primary endpoint of the present study was all-cause mortality. To analyze mortality in detail, secondary endpoints were cardiovascular mortality, HF hospitalization, stroke, and myocardial infarction (MI). CV mortality included death from myocardial infarction (MI), sudden death, stroke, pump failure, pulmonary embolism, and CV procedure-related events. Medication usage data was collected based on a combination of medical record reviews and interviews at baseline visits. The outcomes were monitored through a prespecified period by a clinical endpoint committee at the Brigham and Women's Hospital. The patients were assessed every 4 months during the first year of the study and every 6 months thereafter.

All participants in ACCORD trials would be instructed to attend the clinic following an overnight fast. During the visit, the eligibility status was confirmed. If eligible, the baseline history and physical exam would be obtained by a trained technician. Waist circumference was measured using metallic measuring tapes according to the NHANES (the National Health and Nutrition Examination Survey) III protocol (during normal minimal respiration and at the smallest point between the tenth rib and the iliac crest). Abdominal obesity was defined as a waist circumference of ≥102 cm in men and ≥88 cm in women [13].

### 2.3. Statistical Analysis

All statistical analyses were performed using IBM SPSS 25.0 (IBM Corp, Armonk, NY). Baseline characteristics across the quartiles were summarized as frequencies and percentages for categorical variables, and as means (standard deviation) or median (interquartile range (IQR)) for continuous variables, depending upon whether the data were normally distributed (assessed by normal Q–Q plots). Continuous variables were compared using Student's *t*-test, and categorical variables were compared using Chi-square tests. Kaplan-Meier survival curves were constructed for the gender comparison of primary and secondary outcomes, with and without abdominal obesity. Differences in cumulative incidence curves were compared via the log-rank test.

Cox proportional hazard models were used to analyze and compare hazard ratios (HRs) for the primary and secondary outcomes with 95% confidence intervals (CIs). The proportional hazard assumption was examined by graphical methods using the scaled Schoenfeld residuals. Because multicollinearity statistical analyses may yield biased estimates, various analyses employed different models to evaluate the association between abdominal obesity and mortality. In model 1, the following parameters were adjusted: age, race, smoking, alcohol usage, and living status. In model 2, the following parameters were adjusted: age, race, smoking and alcohol consumption, living status, NYHA functional class, blood pressure, heart rate, myocardial infarction, congestive heart failure, COPD, and mean KCCQ overall score. In model 3, the following parameters were adjusted: age, race, smoking and alcohol consumption, living status, NYHA functional class, blood pressure, heart rate, myocardial infarction, congestive heart failure, COPD, Mean KCCQ overall score, creatinine, eGFR, ALT, blood glucose, use of angiotensin-converting enzyme inhibitors or angiotensin II receptor blockers, calcium channel blockers, and antihypertensive and beta-blocker usage.

All-cause mortality was further analyzed according to clinically relevant subgroups: age (<60 or ≥60 years), NYHA functional class (NYHA I and II or NYHA III and IV), hypertension (nonhypertensive or hypertensive), and DM (no DM or DM). To explore effect modification, interactions with abdominal obesity in patients at rest were analyzed among the groups utilizing a multivariable model 3. A two-sided *p* value < 0.05 was considered significant.

## 3. Results

### 3.1. Baseline Characteristics

A total of 3320 participants were included in present study, 2421 patients presented abdominal obesity (female =1396; male =1025). Baseline characteristics are depicted in Table 1. In general, males were younger and had more comorbidities, including coronary artery diseases and interventions (MI, CABG, and angina pectoris), dyslipidemia, diabetes mellitus, COPD, and stroke than females. Males had significantly higher creatinine, eGFR, potassium, and glucose levels and were more likely to be taking beta-blockers, statins, hypoglycemic agents, or CCB than their female counterparts. Females had higher NYHA functional classes (III and IV).

Three self-administered quality of life questionnaires that had been previously validated were used during the study period: KCCQ, EQ-5D, and PHQ. Female participants had lower KCCQ and EQ-5D scores and exhibited more severe depression when compared with males (Table 1).

Echocardiographic data showed that males had higher LV mass index, posterior wall thickness mass, and higher LA enlargement. Females had a worse diastolic function and higher EF than males (Table 2). Baseline characteristics of females and males are depicted in Table S1 and Table S2.

### 3.2. Primary and secondary outcomes

**Figure 1:**
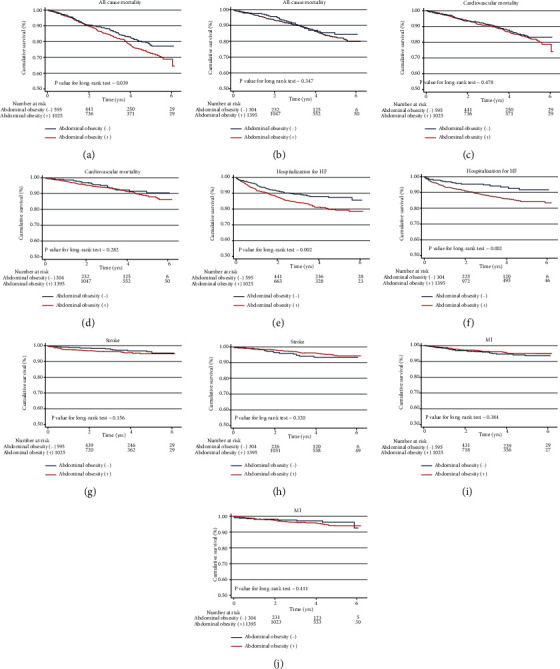
Kaplan-Meier survival curves and cumulative event rates for all-cause mortality in male (a) and female (b); cardiovascular mortality in male (c) and female (d) hospitalization for HF in male (e) and female (f); stroke in male (g) and female (h); MI in male (i) and female (j).

During the follow-up period, 503 patients died. Kaplan-Meier survival curves and cumulative event rates for all-cause, cardiovascular mortality, HF hospitalization, stroke, and MI with and without abdominal obesity are shown in Figure 1 and Table 3, respectively. After multivariable adjustment, abdominal obesity was found to be significantly associated with an increased risk of all-cause mortality in males (model 1, adjusted HR: 1.38; 95% CI: 1.08 to 1.77; *p* = 0.010; model 2, adjusted HR: 1.37; 95% CI: 1.06 to 1.76; *p* = 0.016; and model 3, adjusted HR: 1.32; 95% CI: 1.02 to 1.71; *p* = 0.038) (Table 3). Moreover, abdominal obesity was associated with the risk of hospitalization for HF in males (model 1, adjusted HR: 1.62; 95% CI: 1.20 to 2.18; *p* = 0.001; model 2, adjusted HR: 1.50; 95% CI: 1.11 to 2.04; *p* = 0.009; and model 3, adjusted HR: 1.39; 95% CI: 1.01 to 1.93; *p* = 0.045) and females (model 1, adjusted HR: 1.95; 95% CI: 1.19 to 3.18; *p* = 0.008; model 2, adjusted HR: 1.85; 95% CI: 1.23 to 3.03; *p* = 0.015; and model 3, adjusted HR: 1.15; 95% CI: 1.18 to 3.28; *p* = 0.01) (Table 3). No significant difference was observed in the risk of cardiovascular mortality, stroke, and MI between males and females with abdominal obesity.

### 3.3. Interaction and sensitivity analyses

**Figure 2:**
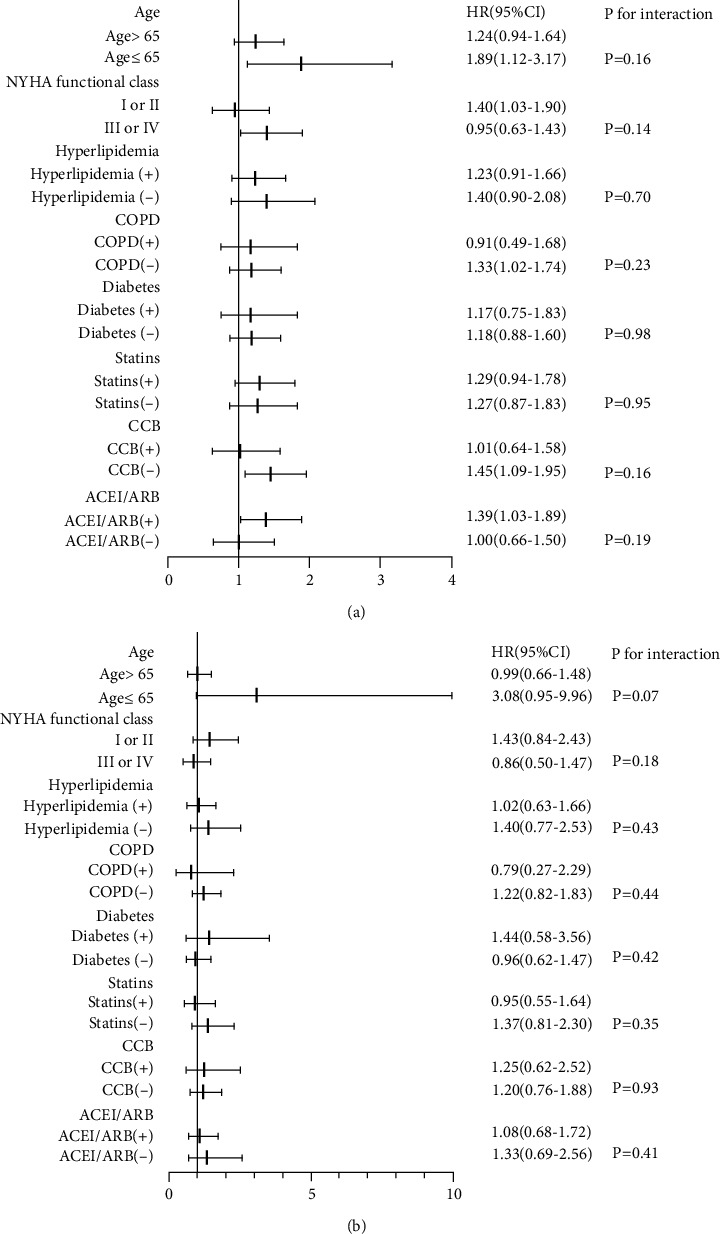
Association between abdominal obesity and all-cause mortality in the subgroups, (a) males and (b) females. COPD: chronic obstructive pulmonary disease; CCB: calcium channel blocker; ACEI: angiotensin-converting enzyme inhibitors; ARB: angiotensin II receptor blockers; NYHA: New York Heart Association.

Figure 2 illustrates the association between abdominal obesity and all-cause mortality in the different subgroups. No interactions were unearthed among abdominal obesity and age, NYHA functional class, hyperlipidemia, COPD, T2DM, or use of CCB, ACE, and statins, in either gender. All-cause mortality in older males was lower in the low NYHA functional group that did not have COPD and were not taking CCB or using ACE inhibitors than those without abdominal obesity.

## 4. Discussion

Our data suggest that abdominal obesity in male but not female patients with HFpEF is linked with higher risks of all-cause mortality. There were no differences in CV mortality, stroke, or MI between males and females. Importantly, abdominal obesity was independently associated with an increased risk of all-cause mortality after adjustment for confounding variables. Given that the pathophysiological mechanisms and effective treatments for HFpEF remain poorly defined [14], the hereby present findings could be an important theoretical support for the formulation of HFpEF treatments that consider patient gender and the correlation with abdominal obesity.

Obesity is highly prevalent in HFpEF patients (>80%) [15–17] and involves unique pathophysiological features. Obesity exerts direct and indirect effects on the progress of HFpEF, including increased myocardial load, worsening of arterial hypertension, and damage of the left ventricular (LV) structure and the diastolic and systolic function [2, 18–20]. Recent research has revealed that fat tissue, in particular, the abdominal fat, is associated with several adverse cardiac functions even in nonobese individuals, independently of BMI [21]. This suggests that not only the amount but also the regional fat distribution may serve as a pivotal predictor in patients with HFpEF. Notably, adipose tissue distribution is known to vary between genders. Men start to lose lean mass after age 50, and women show a similar decline but also an increase in fat mass [22]. In the present study, patients with abdominal obesity were 1025 males (63.27%) and 1396 females (82.12%). There is reason to believe that this divergent fat distribution between genders affects clinical outcomes. Unfortunately, only a few studies have explored gender differences regarding the relationship between abdominal obesity and the prognosis of HFpEF, which seem to be of strong clinical relevance.

In the current study, we utilized waist circumference to measure abdominal obesity and evaluate its relationship with the prognosis of HFpEF. Considering that BMI does not distinguish between fat and lean mass, it appears reasonable to use parameters such as waist circumference, waist-to-hip ratio, and waist-to-height ratio to measure body composition. Waist circumference stands as the most commonly used method [23–25].

In the present study, the male group was younger, with lower blood pressure and prevalence of comorbidities. Males also had lower NYHA functional classification values III-IV and higher class I-II and were more likely to take statins, hypoglycemic agents, and/or beta-blockers. Cumulatively, this suggests that males are diagnosed at a younger age and that comprehensive management treatment plans should be designed early. These plans should include comorbidity-specific treatments, as well as multifactorial lifestyle modification interventions to potentially reduce the burden of HFpEF. Our study showed that females reported a lower quality of life, with worse depression than their male counterparts, which suggests a critical need for treatment adjustments, better management strategies, and appropriate psychological intervention measures.

Echocardiographic findings indicated that female participants had a worse diastolic function and higher EF than males. Given that average age was higher in females than in males, noninvasive measurements of diastolic function in HFpEF patients appear to change with age. At least 1 abnormal diastolic measurement was noted in >90% of those older than 65 years of age [26–28]. Consistent with prior studies, our results indicated that males had greater LV mass index and posterior wall thickness mass. They also showed higher LA enlargement, which was associated with a heightened risk for HF hospitalization or CV death. LA volume is considered a reliable estimator of chronic LV filling pressure to predict adverse outcomes in HFpEF [29, 30].

Our research confirmed that abdominal obesity is an independent risk factor for the prognosis of male patients with HFpEF after a long-term follow-up. Nonetheless, abdominal obesity may not be a reliable predictor of mortality in females with HFpEF. This outcome may be attributed to the “obesity paradox,” where obese patients with HF (especially in some specific subgroups) have a more favorable prognosis than those with healthier weight [31–33]. Epidemiologic studies also found that this paradox appears more frequently in females [11, 34, 35]. Even though the reasons for those findings remain unknown, there is basic scientific research to support this position. For example, Peterson et al. demonstrated that females exhibit greater myocardial fatty acid metabolism, a decrease in metabolism efficiency, and lower myocardial glucose utilization [36]. Ovarian hormones have also been associated with the regulation of myocardial substrate metabolism [37]. Male mice, which are deficient in peroxisome proliferator-activated receptors, have abnormalities in the cardiac lipid metabolism [38]. Furthermore, Pilate et al. suggested that estrogen may have direct effects on myocardial fatty acid metabolism. Early studies with isotope tracer methods exposed that the basal rate of appearance of fatty acids is higher in women probably because men have higher circulating insulin concentrations, which in turn induces greater suppression of lipolysis [39]. Females also have a higher turnover of fatty acids, thereby raising the probability that their hearts are more dependent on fatty acids for energy production. This could potentially explain the survival advantage of obese females [40]. Notably, the regional distribution of adipose tissue may play a critical role in the development of HFpEF among obese individuals. Men generally store excessive fat in a visceral distribution, while women store fat in a peripheral subcutaneous distribution [41]. Visceral adipose tissue (VAT) is a proinflammatory tissue that may increase cardiovascular risk [42]. As obesity progresses, VAT accumulates and secretes proinflammatory cytokines that may lead to microvascular endothelial dysfunction and affect vascular compliance in HFpEF [43, 44]. These processes could account for the increments in male waist circumference.

The present study demonstrated that the rate of hospitalization for HF affected by abdominal obesity was the same in male and female patients and consistent with previous literature. The causes may be associated with insulin resistance, systemic inflammation, neurohormonal activation, or adipokine abnormalities [45, 46]. This finding highlights that abdominal obesity is a major risk factor for hospitalization after HF in both sexes. In addition, CV mortality, stroke, and MI were not significantly influenced by abdominal obesity in either male or female HFpEF patients. Overall, more studies are important to fully understand the connection between abdominal obesity and the prognosis of HFpEF patients, and long-term follow-up is needed to evaluate mortality after particular interventions.

This study has some limitations. First, waist circumference was identified at the onset of the study but was not reevaluated during the follow-up period. Theoretically, cornerstones of pharmacological and nonpharmacological management in HF have been restricting dietary sodium intake, fluid restriction, and diuretic treatment. There would have dynamic changes in waist circumference because of intensified diuretic therapy and nonadherence to fluid restriction. Therefore, we cannot sufficiently exclude the possible effects of reverse causality. To clarify the association, a dynamic approach to the evaluation of changes in waist circumference over a certain period is of great importance. Second, because this study was a post doc analysis of the TOPCAT trial, the association between waist circumference and adverse outcomes might not translate to other HFpEF populations. Although the statistical modeling is multifactor, we acknowledge that there is a potential for residual confounding.

## 5. Conclusion

The findings of the present study suggest that abdominal obesity in male patients with HFpEF is associated with higher risks of all-cause mortality. This outcome was not found in the female population. Our study assists in identifying lifestyle risk factors for HFpEF between genders and should be regarded as a potentially modifiable target for HFpEF prevention.

### 5.1. Clinical Implications

The gender-specific waist circumference cutoff points may guide the initiation of weight control strategies for the prevention of HFpEF and provide targets for such strategies. The results of our study highlight the need for an appropriate disease-specific resource allocation that provides preventive strategies in the HFpEF population. It may be useful to implement gender-specific preventive strategies and management programs in the HFpEF population. Overall, further studies should be undertaken to elucidate the detailed mechanisms underlying the association between abdominal obesity and adverse outcomes in male HFpEF populations.

## Figures and Tables

**Table 1:** Demographic and clinical characteristics.

Characteristics	All (*n* = 3320)	Male (*n* = 1620)	Female (*n* = 1700)	*p* value
Abdominal obesity no. (%)	2421 (72.92%)	1025 (63.27%)	1396 (82.12%)	<0.001
Age (year, mean ± SD)	68.51 ± 9.46	67.71 ± 9.60	69.28 ± 9.46	<0.001
Race no. (%)				
White	2,990 (90.06%)	1,491 (92.04%)	1,499 (88.18%)	<0.001
Black	253 (7.62%)	89 (5.49%)	164 (9.65%)	<0.001
Other	67 (2.02%)	31 (1.91%)	36 (2.12%)	0.676
BMI (kg/m^2^, mean ± SD)				
<18.5	14 (0.42%)	9 (0.56%)	5 (0.29%)	0.371
18.5-24.9	409 (12.32%)	176 (10.86%)	233 (13.71%)	0.017
25-29.9	1,074 (32.35%)	588 (36.30%)	486 (28.59%)	<0.001
≥ 30.0	1,823 (54.91%)	847 (52.28%)	976 (57.41%)	0.003
Heart rate (mean ± SD)	69.90 ± 10.32	68.63 ± 10.50	69.16 ± 10.15	0.145
Blood pressure(mm/Hg, mean ± SD)				
SBP	129.27 ± 13.86	128.00 ± 13.60	130.49 ± 14.00	<0.001
DBP	75.98 ± 10.57	75.49 ± 10.46	76.44 ± 10.66	0.010
NYHA functional classification no. (%)				0.021
I and II	2,250 (67.77%)	1,129 (69.69%)	1,121 (65.94%)	
III and IV	1,070 (32.23%)	491 (30.31%)	579 (34.06%)	
Comorbidities no. (%)				
Hypertension	3,040 (91.57%)	1,454 (89.75%)	1,586 (93.29%)	<0.001
Hospitalization for heart failure	2,412 (72.65%)	1,175 (72.53%)	1,237 (72.76%)	0.880
MI	879 (26.48%)	554 (34.20%)	325 (19.12%)	<0.001
CABG	428 (12.89%)	311 (19.20%)	117 (6.88%)	<0.001
Diabetes mellitus	1,068 (32.17%)	554 (34.20%)	514 (30.24%)	0.015
Dyslipidemia	2,001 (60.27%)	1,040 (64.20%)	961 (56.53%)	<0.001
COPD	375 (11.30%)	229 (14.14%)	146 (8.59%)	<0.001
Stroke	253 (7.62%)	121 (7.47%)	132 (7.76%)	0.748
Laboratory (mean ± SD)				
Creatinine(mg/dL)	1.09 ± 0.30	1.19 ± 0.30	1.00 ± 0.25	<0.001
eGFR (ml/min/1.73 m^2^)	67.78 ± 20.06	70.69 ± 19.89	65.02 ± 19.83	<0.001
Sodium (mmol/L)	141.28 ± 4.24	141.13 ± 4.23	141.42 ± 4.25	0.050
Potassium (mmol/L)	4.26 ± 0.45	4.28 ± 0.44	4.24 ± 0.45	0.020
ALT (UL)	25.17 ± 14.36	26.10 ± 15.05	24.30 ± 13.63	0.003
Glucose (mg/dL)	115.23 ± 47.80	117.12 ± 49.95	113.44 ± 46.63	0.030
BNP (pg/ml)	382.10 ± 428.91	387.09 ± 394.04	376.60 ± 464.88	0.746
NT-proBNP (pg/ml)	1511.25 ± 2188.05	1457.79 ± 2011.81	1563.17 ± 2348.68	0.548
Medications no. (%)				
ACE-I/ARB	2,807 (84.55%)	1,369 (84.51%)	1,438 (84.59%)	0.981
Diuretic	2,704 (81.45%)	1,300 (80.25%)	1,404 (82.59%)	0.089
Beta-blockers	2,584 (77.83%)	1,288 (79.51%)	1,296 (76.24%)	0.021
Aspirin	2,180 (65.66%)	1,084 (66.91%)	1,096 (64.47%)	0.132
Statin	1,724 (51.93%)	944 (58.27%)	780 (45.88%)	<0.001
Calcium channel blocker	1,242 (37.41%)	575 (35.49%)	667 (39.24%)	0.027
Hypoglycemic agent	915 (27.56%)	480 (29.63%)	435 (25.59%)	0.009
Currently smoke no. (%)	352 (10.60%)	260 (16.05%)	92 (5.41%)	<0.001
Alcohol drinks in the past weeks no. (%)				
None	2,595 (78.16%)	1,104 (68.15%)	1,491 (87.71%)	<0.001
1–4	555 (16.72%)	382 (23.58%)	173 (10.18%)	<0.001
5–10	119 (3.58%)	90 (5.56%)	29 (1.71%)	<0.001
>11	51 (1.54%)	44 (2.72%)	7 (0.41%)	<0.001
Mean KCCQ overall score (mean ± SD)	54.91 ± 20.36	58.53 ± 20.79	51.46 ± 19.33	<0.001
PHQ no. (%)				0.231
<10	995 (29.97%)	539 (33.27%)	456 (26.82%)	<0.001
≥10	344 (10.36%)	171 (10.56%)	173 (10.18%)	0.720

Values are mean ± SD or no. (%). BMI: body mass index; SBP: systolic blood pressure; DBP: diastolic blood pressure; MI: myocardial infarction; COPD: chronic obstructive pulmonary diseases; CABG: coronary artery bypass graft; eGFR: estimated glomerular filtration rate; BUN: blood urea nitrogen; NT-proBNP: N-terminal pro-BNP; ACE-I: angiotensin-converting enzyme inhibitors; ARB: angiotensin II receptor blockers; NYHA: New York Heart Association.

**Table 2:** Echocardiographic comparisons.

	All (*n* = 3320)	Male (*n* = 1620)	Female (*n* = 1700)	*p* Value
LV structure (mean ± SD)				
End-diastolic dimension, cm	4.81 ± 0.57	4.99 ± 0.57	4.60 ± 0.51	<0.001
End-diastolic diameter index, cm/m^2^	2.41 ± 0.40	2.34 ± 0.37	2.45 ± 0.37	<0.001
End-diastolic volume, mL	99.32 ± 33.68	112.42 ± 33.92	85.72 ± 27.49	<0.001
End-diastolic volume index, mL/m^2^	49.75 ± 15.74	53.49 ± 15.90	45.86 ± 14.61	<0.001
End-systolic dimension, cm	3.37 ± 0.51	3.54 ± 0.52	3.18 ± 0.44	<0.001
End-systolic diameter index, cm/m^2^	1.69 ± 0.30	1.67 ± 0.31	1.70 ± 0.28	0.165
End-systolic volume, mL	41.55 ± 20.29	48.81 ± 21.52	34.22 ± 15.70	<0.001
End-systolic volume index, mL/m^2^	20.83 ± 9.94	23.19 ± 10.63	18.43 ± 8.55	<0.001
Septum wall thickness, cm	1.20 ± 0.20	1.25 ± 0.20	1.14 ± 0.19	<0.001
Posterior wall thickness, cm	1.16 ± 0.19	1.20 ± 0.19	1.11 ± 0.18	<0.001
LV mass, g	219.03 ± 68.84	244.78 ± 68.16	191.33 ± 58.02	<0.001
LV mass index, g/m^2^	108.28 ± 30.30	114.52 ± 30.21	101.58 ± 28.97	<0.001
EF (mean ± SD)	58.97 ± 7.87	57.73 ± 8.16	60.29 ± 7.33	<0.001
LV diastolic properties				
Diastolic dysfunction no. (%)				
Normal	142 (4.28%)	82 (5.06%)	60 (3.53%)	0.029
Mild	102 (3.07%)	39 (2.41%)	63 (3.71%)	0.030
Moderate	145 (4.37%)	57 (3.52%)	88 (5.18%)	0.019
Severe	43 (1.30%)	22 (1.36%)	21 (1.24%)	0.755
E, cm/s (mean ± SD)	86.23 ± 29.61	86.05 ± 29.89	86.42 ± 29.36	0.871
A, cm/s (mean ± SD)	74.22 ± 24.52	69.26 ± 23.53	78.46 ± 24.60	<0.001
E/A (mean ± SD)	1.23 ± 0.67	1.26 ± 0.67	1.20 ± 0.68	0.275
E/E′ lateral (mean ± SD)	11.61 ± 5.79	11.02 ± 5.23	12.13 ± 6.20	0.045
E/E′ septal (mean ± SD)	15.50 ± 6.87	15.32 ± 6.91	15.68 ± 6.84	0.581
E deceleration time, ms (mean ± SD)	208.49 ± 66.15	204.51 ± 67.14	212.53 ± 64.99	0.120
Left atrial area, cm^2^ (mean ± SD)	19.43 ± 5.43	20.23 ± 5.74	18.62 ± 4.99	<0.001
Pulmonary vascular and right ventricle (mean ± SD)				
TR jet velocity, m/s	277.98 ± 45.41	271.55 ± 42.09	283.59 ± 47.50	0.008
PVR, wood units	1.88 ± 0.87	1.86 ± 0.56	1.90 ± 1.06	0.686
RV FAC (%)	0.48 ± 0.08	0.47 ± 0.07	0.49 ± 0.08	0.001

**Table 3:** The risk of primary and second outcomes in HFpEF patients among genders.

	Abdominal obesity in male			Abdominal obesity in female		
	Yes (*n* = 1025)	No (*n* = 595)	*p* Value	Yes (n =1396)	No (*n* = 304)	*p* Value
*All-cause mortality*						
Cases/*n*	202 (1025)	96 (595)		173 (1396)	32 (304)	
Unadjusted HR (95% CI)	1.29 (1.01 1.64)	1.00 (ref)	0.039	1.20 (0.82 1.74)	1.00 (ref)	0.347
Model 1: adjusted HR (95% CI)	1.38 (1.08 1.77)	1.00 (ref)	0.001	1.28 (0.87 1.88)	1.00 (ref)	0.210
Model 2: adjusted HR (95% CI)	1.37 (1.06 1.76)	1.00 (ref)	0.016	1.20 (0.81 1.76)	1.00 (ref)	0.358
Model 3: adjusted HR (95% CI)	1.32 (1.02 1.71)	1.00 (ref)	0.038	1.15 (0.77 1.71)	1.00 (ref)	0.482
*Cardiovascular mortality*						
Cases/*n*	123 (1025)	68 (595)		112 (1396)	19 (304)	
Unadjusted HR (95% CI)	1.11 (0.83 1.50)	1.00 (ref)	0.478	1.31 (0.80 2.12)	1.00 (ref)	0.282
Model 1: adjusted HR (95% CI)	1.19 (0.88 1.61)	1.00 (ref)	0.255	1.36 (0.83 2.23)	1.00 (ref)	0.221
Model 2: adjusted HR (95% CI)	1.20 (0.88 1.63)	1.00 (ref)	0.255	1.21 (0.73 1.20)	1.00 (ref)	0.459
Model 3: adjusted HR (95% CI)	1.15 (0.84 1.57)	1.00 (ref)	0.397	1.15 (0.69 1.92)	1.00 (ref)	0.589
Hospitalization for HF						
Cases/*n*	162 (1025)	62 (595)		167 (1396)	18 (304)	
Unadjusted HR (95% CI)	1.60 (1.20 2.15)	1.00 (ref)	0.002	2.12 (1.31 3.45)	1.00 (ref)	0.002
Model 1: adjusted HR (95% CI)	1.62 (1.20 2.18)	1.00 (ref)	0.001	1.95 (1.19 3.18)	1.00 (ref)	0.008
Model 2: adjusted HR (95% CI)	1.50 (1.11 2.04)	1.00 (ref)	0.009	1.85 (1.23 3.03)	1.00 (ref)	0.015
Model 3: adjusted HR (95% CI)	1.39 (1.01 1.93)	1.00 (ref)	0.045	1.15 (1.18 3.28)	1.00 (ref)	0.010
*Stroke*						
Cases/*n*	36 (1025)	14 (595)		47 (1396)	14 (304)	
Unadjusted HR (95% CI)	1.56 (0.84-2.90)	1.00 (ref)	0.156	0.74 (0.41-1.34)	1.00 (ref)	0.320
Model 1: adjusted HR (95% CI)	1.53 (0.82-2.86)	1.00 (ref)	0.180	0.70 (0.38-1.28)	1.00 (ref)	0.243
Model 2: adjusted HR (95% CI)	1.50 (0.79-2.84)	1.00 (ref)	0.212	0.65 (0.35-1.20)	1.00 (ref)	0.171
Model 3: adjusted HR (95% CI)	1.42 (0.74-2.72)	1.00 (ref)	0.297	0.63 (0.33-1.17)	1.00 (ref)	0.143
*MI*						
Cases/*n*	36 (1025)	27 (595)		54 (1396)	9 (304)	
Unadjusted HR (95% CI)	0.80 (0.49-1.32)	1.00 (ref)	0.384	1.34 (0.66-2.72)	1.00 (ref)	0.411
Model 1: adjusted HR (95% CI)	0.89 (0.53-1.47)	1.00 (ref)	0.642	1.30 (0.64-2.66)	1.00 (ref)	0.470
Model 2: adjusted HR (95% CI)	0.85 (0.50-1.44)	1.00 (ref)	0.562	1.04 (0.50-2.14)	1.00 (ref)	0.918
Model 3: adjusted HR (95% CI)	0.80 (0.47-1.36)	1.00 (ref)	0.414	1.23 (0.57-2.68)	1.00 (ref)	0.591

Model 1 adjusted for age, race, smoking and alcohol consumption, and living status. Model 2 adjusted for age, race, smoking alcohol consumption, living status, NYHA functional class, blood pressure, heart rate, myocardial infarction, congestive heart failure, COPD, and mean KCCQ overall score. Model 3 adjusted for age, race, smoking and alcohol consumption, living alone, NYHA functional class, blood pressure, heart rate, myocardial infarction, congestive heart failure, COPD, mean KCCQ overall score, creatinine, eGFR, ALT, blood glucose, use of calcium channel blockers, ACEI/ARB, antihypertensive, and beta-blockers.

## Data Availability

The datasets used and analyzed during the current study are available from TOPCAT Research Materials obtained from the National Heart, Lung, and Blood Institute (NHLBI) Biologic Specimen and Data Repository Information Coordinating Center and does not necessarily reflect the opinions or views of the TOPCAT or the NHLBI.

## Supplementary Materials

Supplementary Materials Table S1: demographic and clinical characteristics of female patients. Table S2: demographic and clinical characteristics of male patients.
