# Supplementary material for: Abdominal Obesity Is Associated with an Increased Risk of All-Cause Mortality in Males but Not in Females with HFpEF
Source: Cardiovasc Ther. 2022 Apr 9;2022:2950055. doi: 10.1155/2022/2950055 (PMC9013300; doi:10.1155/2022/2950055)
Supplement: Supplementary Materials — Table S1: demographic and clinical characteristics of female patients. Table S2: demographic and clinical characteristics of male patients. [file 2950055.f1.pdf]

Table S1 Demographic and Clinical Characteristics of Female Patients

Characteristics	Abdominal Obesity		
	No(n=304)	Yes(n=1396)	p Value
**Age (year, mean ± SD)**	69.96±9.77	69.14±9.39	0.181
**Race No. (%)**			
White	283(93.09%)	1,216(87.11%)	0.003
Black	12(3.95%)	152(10.89%)	<0.001
Other	10(3.29%)	33(2.36%)	0.352
**BMI, (kg/m^2, mean ± SD)**			
<18.5	5(1.64%)	0(0)	<0.001
18.5-24.9	163(53.62%)	70(5.01%)	<0.001
25-29.9	112(36.84%)	374(26.79%)	<0.001
>=30.0	24(29.61%)	953(68.27%)	<0.001
**Heart rate (mean ± SD)**	68.65±9.45	69.27±10.30	0.311
**Blood pressure（mm/Hg, mean ± SD）**			
SBP	128.31±13.09	130.97±14.15	0.002
DBP	75.81±10.78	76.57±10.63	0.266

**NYHA functional classification No. (%)**			0.071
I & II	214(70.39%)	907(64.97%)	
III & IV	90(29.61%)	489(35.03%)	
**Comorbidities No. (%)**			
Hypertension	273(89.80%)	1,313(94.05%)	0.007
Hospitalization for heart failure	215(70.72%)	1,022(73.21%)	0.378
MI	46(15.13%)	279(19.99%)	0.051
CABG	17(5.59%)	100(7.16%)	0.327
Diabetes mellitus	39(12.83%)	475(34.03%)	<0.001
Dyslipidemia	139(45.72%)	822(58.88%)	<0.001
COPD	17(5.59%)	129(9.24%)	0.040
Stroke	15(4.93%)	117(8.38%)	0.042
**Laboratory (mean ± SD)**			
Creatinine(mg/dL)	0.93±0.23	1.01±0.26	<0.001
eGFR (ml/min/1.73 m^2)	68.64±23.15	64.23±18.95	0.002
Sodium(mmol/L)	141.73±4.98	141.35±4.07	0.224
Potassium(mmol/L)	4.24±0.46	4.24±0.45	0.976
ALT(UL)	22.87±12.19	24.61±13.91	0.023

Glucose(mg/dL)	100.50±35.23	116.26±48.32	<0.001
BNP (pg/ml)	468.30± 50.07	359.67± 389.90	0.308
NT-proBNP (pg/ml)	2031± 2413.03	1446.21±2322.48	0.086
**Medications No. (%)**			
ACE-I/ARB	245(80.59%)	1,193(85.46%)	0.033
Diuretic	217(71.38%)	1,187(85.035)	<0.001
Beta blockers	228(75.00%)	1,068(76.50%)	0.577
Aspirin	199(65.46%)	897(64.26%)	0.691
Statin	112(36.84%)	668(47.85%)	<0.001
Calcium channel blocker	98(32.24%)	569(40.76%)	0.006
Hypoglycemic agent	32(10.53%)	403(28.87%)	<0.001
**Currently smoke No. (%)**	27(8.88%)	65(4.66%)	0.003
**Alcohol drinks in the past weeks No. (%)**			
None	261(85.86%)	1230(88.11%)	0.278
1–4	34(11.18%)	139(9.96%)	0.521
5–10	8(2.63%)	21(1.50%)	0.169
>11	1(0.33%)	6(0.43%)	0.803
**Mean KCCQ overall score (mean ± SD)**	54.22±52.21	50.86±49.83	0.004

PHQ No. (%)			0.004
<10	72(23.68%)	384(27.51%)	
>=10	18(5.92%)	155(11.10%)	

Table S2 Demographic and Clinical Characteristics of Male Patients

**Characteristics**	**Abdominal Obesity**		
	**No(n=595）**	**Yes(n=1025)**	**p Value**
**Age (year, mean ± SD)**	68.50± 9.98	67.25±9.35	0.013
**Race No. (%)**			
White	549(92.27%)	942(91.90%)	0.793
Black	26(4.37%)	63(6.15%)	0.130
Other	22(3.70%)	20(1.95%)	0.033
**BMI, (kg/m^2, mean ± SD)**			
<18.5	9(1.51%)	0(0)	<0.001
18.5-24.9	163(27.39%)	13(1.27%)	<0.001
25-29.9	339(56.97%)	249(24.29%)	<0.001
>=30.0	84(14.12%)	763(74.44%)	<0.001
**Heart rate (mean ± SD)**	67.69±9.99	69.18±10.75	0.005
**Blood pressure（mm/Hg, mean ± SD）**			
SBP	127.16±13.09	128.47±13.87	0.058
DBP	76.13±9.88	75.12± 10.77	0.057
**NYHA functional classification No. (%)**			<0.001

I & II	457(76.81%)	672(65.56%)	
III & IV	138(23.19%)	353(34.44%)	
**Comorbidities No. (%)**			
Hypertension	520(87.39%)	934(91.12%)	0.017
Hospitalization for heart failure	432(72.61%)	743(72.49%)	0.959
MI	218(36.64%)	336(32.78%)	0.115
CABG	101(16.97%)	210(20.49%)	0.084
Diabetes mellitus	117(19.66%)	437(42.63%)	<0.001
Dyslipidemia	352(59.16%)	688(67.12%)	0.001
COPD	62(10.42%)	167(16.29%)	0.001
Stroke	43(7.23%)	78(7.61%)	0.778
**Laboratory (mean ± SD)**			
Creatinine(mg/dL)	1.15±0.28	1.21±0.01	<0.001
eGFR (ml/min/1.73 m^2)	72.76±20.52	69.49±19.42	0.002
Sodium (mmol/L)	141.07±4.26	141.17±4.21	0.662
Potassium (mmol/L)	4.30±0.43	4.26±0.44	0.082
ALT (UL)	24.68±13.32	26.91±15.92	0.003
Glucose (mg/dL)	107.19±33.71	122.87±55.11	<0.001

BNP (pg/ml)	440.97±481.97	364.44±349.15	0.132
NT-proBNP (pg/ml)	1813.76± 2408.90	1224.97± 1669.34	0.020
**Medications No. (%)**			
ACE-I/ARB	494(83.04%)	875(85.37%)	0.193
Diuretic	429(72.10%)	871(84.98%)	<0.001
Beta blockers	478(80.34%)	810(79.02%)	0.553
Aspirin	407(68.40%)	677(66.05%)	0.345
Statin	312(52.44%)	632(61.66%)	<0.001
Calcium channel blocker	195(32.77%)	380(37.07%)	0.079
Hypoglycemic agent	95(15.97%)	385(37.56%)	<0.001
**currently smoke No. (%)**	116(19.5%)	144(14.05%)	0.004
**Alcohol drinks in the past weeks No. (%)**			
None	392(65.88%)	712(69.46%)	0.136
1–4	150(25.21%)	232(22.63%)	0.239
5–10	37(6.22%)	53(5.17%)	0.375
>11	16(2.69%)	28(2.73%)	0.959
**Mean KCCQ overall score (mean ± SD)**	61.15±19.90	57.00±21.15	<0.001
PHQ No. (%)			<0.001

<10	157(26.39%)	382(37.27%)
>=10	38(6.39%)	133(12.98%)
